# Household Savings and Negative Interest Rates

**DOI:** 10.1007/s11294-023-09870-1

**Published:** 2023-04-12

**Authors:** Klaas Staal

**Affiliations:** Karlstad Business School, Karlstad University, Universitetsgatan 2, 65 188 Karlstad, Sweden

**Keywords:** Household savings rate, Savings’ determinants, Negative interest rates, Panel data, F31, F32, F36

## Abstract

This paper analyses determinants of household savings in a model based on an extension of the disequilibrium savings theory. These extensions follow from the life-cycle, permanent-income and Ricardian-equivalence theories. Based on panel data of 20 countries from the period 2000–2020, fixed-effect least squares estimation procedures are used. The analysis provides evidence that negative interest rates lead to a statistically and economic significant increase in savings. This implies that stimulating household consumption with a monetary policy of negative interest rates is counter-productive. The positive effect of income uncertainty and lagged saving rates gets smaller for negative interest rates, weakening the support for the disequilibrium-savings theory. Larger government deficits increase savings even more when rates are negative, strengthening the Ricardian equivalence effect. The effect of negative interest on the predictions of the life-cycle and permanent-income theories is mixed.

## Introduction

National investments are identical to national savings in a closed economy. An increase in savings thus increases investments, and a higher capital stock increases the standard of living. In an open economy, due to the international mobility of capital, the relationship between national savings and investments is weaker, but Weil (2009, p. 69) showed that national investments still significantly depend on national savings. This explains why policy makers have a keen interest in household savings decisions. Therefore, the focus of this paper is on the determinants of household savings decisions, and more specifically on the effects of negative interest rates. According to Brandao-Marques et al. (2021), there are no empirical studies focusing on the effects of negative interest on household savings behavior. Indeed, to my knowledge, this paper is one of very first to do so.

One of the responses of monetary policy makers to the global financial crisis and subsequently the coronavirus disease 2019 (COVID-19) (World Health Organization 2022) pandemic is the introduction of negative interest rate policy (NIRP, see, e.g., Claeys 2021). Indeed, NIRPs remain controversial and one of the concerns is that a NIRP could lead to non-linear effects of monetary policy. The evidence presented in this paper suggests that a NIRP leads to a statistically and economically significant increase in household savings. A further contribution is a more detailed analysis of the effects of NIRP on the effects of the specific determinants of household saving behavior. The detailed analysis gives insights regarding which economic theory increases or loses its predictive power in times of NIRPs. This makes the presented findings relevant for policy makers and for a better economic understanding of negative interest rates.

Deaton (1977) presented the disequilibrium-savings theory of savings. This theory predicts that unanticipated income changes, unanticipated inflation effects, and previous saving levels have a positive effect on household savings. Friedman (1957) introduced the permanent-income theory, while Modigliani and Brumberg (1954) and Ando and Modigliani (1963) described the life-cycle theory. The latter two theories can be used to predict a positive effect of income uncertainty, and negative effects of social security, the old-age dependency ratio, the participation rate of the elderly and health expenditure on households’ savings. Finally, Barro (1974) provided a theoretical foundation for the Ricardian equivalence proposition. This proposition maintains that government deficits have a positive effect on household savings.

This paper analyzes determinants of savings behavior using a panel of 20 countries. The data for the years 2000 to 2020 are from the Organisation for Economic Co-operation and Development (OECD) website. The regression analysis uses a fixed-effects least-squares approach with household savings ratio as the dependent variable. The main conclusions are that the positive effect of income uncertainty and lagged saving rates gets smaller for negative interest rates, weakening the support for the disequilibrium-savings theory. Larger government deficits increase savings even more when rates are negative, strengthening the Ricardian equivalence effect. The predictions are also considered based on the permanent-income and life-cycle theories. On the one hand, negative interest rates weaken the predicted negative effects of health expenditure and weaken the predicted positive effect of income uncertainty. On the other hand, negative interest rates may strengthen the predicted negative effect of social security on household savings. This implies that negative interest rates have a mixed effect on the empirical validity of the permanent-income and life-cycle theories.

Instead of a comprehensive review of the vast empirical literature on household savings behavior, this literature review is limited to studies based on an approach similar to the one used in this paper. Feldstein (1974, 1977, 1980) and Fredriksson and Staal (2021) presented evidence that social security depresses household savings, as predicted by the permanent-income and life-cycle theories. However, Koskela and Virén (1983) did not find a significant effect of social security, but presented evidence supporting the disequilibrium-savings theory. The findings presented in Nicolescu-Aron and Mihăescu (2012), Aizenman et al. (2019), and Fredriksson and Staal (2021) support the latter theory, indicating that unanticipated income, unanticipated inflation and the perceived optimal savings ratio have positive effects on savings. Finally, El Mekkaoui de Freitas and Martins (2014) and Aizenman et al. (2019) provided evidence for the Ricardian equivalence proposition.

## Theory and Hypotheses

Disequilibrium-savings theory (Deaton 1977) assumes that individuals have incomplete information on prices. According to the theory, individuals also cannot distinguish between relative and absolute price changes. Consequently, actual real income deviates from anticipated real income and individuals save this deviation. Thus, unanticipated income changes have a predicted positive effect on the savings ratio. Inflation has an additional effect on savings. Individuals have the potential to find substitutes for goods that are increasing in price faster than other items. Thus, the avoided expenses are added to savings, so unanticipated inflation is expected to have a positive effect on the savings ratio. Lacking complete information on prices, consumers must update their price information and reconsider their savings decisions. However, previous savings are signaling the approximate preferred savings ratio. Thus, the lagged savings ratio is expected to affect savings positively. As in Koskela and Virén (1983) and Fredriksson and Staal (2021), the disequilibrium-savings theory is the foundation for the estimated savings function. In addition to the unanticipated income effect due to inflation, as well as the inflation effect on savings and the lagged savings ratio motivated by this theory, the analysis also considers interest rates, income uncertainty, social security spending, the old-age dependency ratio, the participation rate of the elderly, government surplus, and health expenditures as explanatory variables for saving decisions.

Changes in interest rates have two opposite effects on savings. The substitution effect increases savings in the case of a rate increase, due to an increase in the returns on savings. However, the income effect decreases savings when rates increase, as less savings are necessary to maintain the same standard of living in the future. The more common finding in the empirical literature is that the substitution effect outweighs the income effect, so that interest rates have an expected positive effect on the savings ratio. The permanent-income theory (Friedman 1957) predicts that individuals increase cautionary savings to compensate for income decreases. These savings help to smooth consumption expenditures. Therefore, income uncertainty is expected to positively affect the savings ratio. The life-cycle theory (Ando and Modigliani, 1963; Modigliani and Brumberg, 1954) also maintains that individuals use savings to smooth consumption expenditures, and that working individuals, therefore, save while retirees dissave. However, these savings can be crowded out by social security spending. Pay-as-you-go social security spending thus has an anticipated negative effect on the savings ratio. Dissaving by retirees also implies that the dependency ratio, measuring the share of the elderly in a population, is expected to have a negative effect on the savings ratio. The more the elderly participate in the labor force, the higher their income and the less savings needed to smooth consumption during retirement. The participation rate affects the savings ratio in a prospective negative way. Ricardian equivalence predicts that a government surplus has a negative effect on the savings ratio (see, e.g., Barro 1974 for a theoretical formalization). Finally, public health expenditure decreases the need for precautionary savings for consumption smoothing, a crowding-out effect, and thus is expected to have a negative effect on the savings ratio.

## Data

The analysis uses OECD data with observations for 20 countries^1^ from 2000 to 2020. The choice of countries and time period were based on the availability of the data on OECD’s website. Table 1 presents the descriptive statistics.

**Table 1:** Descriptive statistics

	# Obs	Mean	St. Dev	Min	Max
Household savings rate (dependent variable, % of disposable income)	420	5.717	4.234	-5.693	21.610
Disposable income (USD per capita)	420	27,117	7,758	9,058	58,653
Expenditure deflator index (national reference year = 1)	420	0.914	0.107	0.529	1.165
Long-term interest rate (% per year)	418	3.348	1.989	-0.511	10.547
Short-term interest rate (% per year)	413	2.052	2.146	-0.695	11.388
Unemployment rate (% of total labor force)	420	7.641	3.609	2.016	26.116
Social security spending (% of GDP)	420	14.152	3.386	6.505	24.037
Old-age dependency ratio (ratio of 65+ to 20–64)	420	27.528	4.864	17.258	39.826
Participation rate 65+	420	7.044	4.949	0.984	20.156
Government surplus (% of GDP)	420	-2.034	4.782	-32.118	18.637
Health expenditure (% of GDP)	420	7.065	1.593	4.323	15.949

Source: Own calculations based on OECD (2023a, 2023b, 2023c, 2023d, 2023e, 2023f, 2023g, 2023h, 2023i, 2023j, 2023k) data from 2000–2020

Household savings behavior is the dependent variable in the analysis. OECD (2023a, p. 1) measures the household net saving rate as “household net disposable income plus the adjustment for the change in pension entitlements less household final consumption expenditure.” These savings are expressed as a percentage of household income.

The other variables in Table 1 are used for the regressors in the analysis. Disposable income (OECD 2023b) comprises wages, and income from investment and self-employment pensions and other social benefits, less any payments of taxes, social insurance contributions and interest on financial liabilities. It is measured in United States dollar (USD) per capita at current prices and purchasing power parities (PPPs). OECD (2023c) provides the private final consumption-expenditure deflator index, with different reference years when the index is equal to one. Long-term interest rates (OECD 2023d) are those for government bonds maturing in ten years, while short-term rates (OECD 2023e) are generally averages of daily three-month money market interest rates. Both rates are measured as a percentage per annum. There are 13 negative long-term and 78 negative short-term interest rates. The long-term interest rates were missing for Czechia and Slovakia for the first year in the sample. However, this is inconsequential, due to the lagging of other variables in the analysis. The Hungarian short-term interest rates were missing seven times. Consequently, Hungary is missing in the part of the analysis that is using the short-term, instead of the long-term, interest rates. The unemployment rate (from OECD 2023f) represents the share of a country’s citizens of working age who are without work, are available for work, and have taken specific steps to find work. The rate is expressed as a percentage of the total labor force. Social security spending encompasses social benefits in cash measured as a percentage of gross domestic product (GDP) (OECD 2023g). The quotient of the population 65+ to the population 20–64 is the old-age dependency ratio (OECD 2023h), while the quotient of the individuals 65+ who are in the labor force to all individuals 65+ is the participation rate of the 65+ (OECD 2023i). Government surplus measures, as a percentage of GDP, by how much a government’s income is larger than its expenditure (OECD 2023j). Health spending, also as a percentage of GDP (OECD 2023k), measures the final consumption of health care goods and services, financed with government spending and compulsory health insurance.

## Methodology

Consider two main specifications in the analysis of household savings decisions. Each specification uses either the constant-expectation or the static-expectation hypothesis. Moreover, the use of either long-term or short-term interest rates implies that there are two estimates for each specification of a regression model.

The constant-expectation hypothesis for the rates of change in real income and inflation is the basis for the regression model in Eq. (1).

1 $${\left(\frac{\mathrm{s}}{\mathrm{y}}\right)}_{\mathrm{i}\mathrm{t}}={\upbeta}_1\Delta \log {\mathrm{Y}}_{\mathrm{i}{t}}+{\upbeta}_2\Delta \log {\mathrm{P}}_{\mathrm{i}\mathrm{t}}+{\upbeta}_3{\left(\frac{\mathrm{s}}{\mathrm{y}}\right)}_{\mathrm{i,t}-\mathrm{1}}+{\upbeta}_4{\mathrm{R}}_{\mathrm{i}\mathrm{t}}+{\upbeta}_5\Delta {\mathrm{U}}_{\mathrm{i}\mathrm{t}}+{\upbeta}_6{\mathrm{SS}}_{\mathrm{i}\mathrm{t}}+{\upbeta}_7{\mathrm{OLD}}_{\mathrm{i}\mathrm{t}} +{\upbeta}_8\mathrm{P}{\mathrm{R}}_{\mathrm{i}\mathrm{t}}+{\upbeta}_9{\mathrm{GS}}_{\mathrm{i}\mathrm{t}}+{\upbeta}_{10}{\mathrm{HS}}_{\mathrm{i}\mathrm{t}}+\sum\limits_{\mathrm{j}=1}^{20}{\mathrm{d}}_{\mathrm{j}}{\mathrm{D}}_{\mathrm{j}}+{\mathrm{u}}_{\mathrm{1},\mathrm{it}}.$$

The regressand ${\left(s/y\right)}_{it}$ is the (net) household savings for country $i$ in year $t$ (OECD 2023a). The regressor ${\Delta logY}_{it}$ denotes the unanticipated income growth, calculated as the first difference of the log of income, with income from OECD (2023b). Unanticipated inflation, ${\Delta logP}_{it}$, is the first difference of the log of the implicit expenditure deflator, based on the private final consumption-expenditure deflator index (OECD 2023c). The real rate of interest, ${R}_{it}$, is either the long-run (OECD 2023d) or the short-run (OECD 2023e) nominal interest rate. The approximation of income uncertainty, ${\Delta U}_{it}$, is the first difference of the unemployment rate (OECD 2023f). $S{S}_{it}$ denotes social security benefits as a percentage of GDP (OECD 2023g), $OL{D}_{it}$ is the old-age dependency ratio (OECD 2023h), and $P{R}_{it}$ is the participation rate of the people over 65 (OECD 2023i). ${GS}_{it}$ is the government budget surplus (positive) or deficit (negative) as a percentage of GDP (OECD 2023j). ${HS}_{it}$ is health spending, also as a percentage of GDP (OECD 2023k). Finally, each country $j$ has a dummy ${D}_{j}$ that is 1 for country $j$ and 0 otherwise.

The static-expectation hypothesis for the rates of change in real income and inflation is the basis for the regression model depicted by Eq. (2)

2 $${\left(\frac{s}{y}\right)}_{it}={\beta }_{1}\Delta \Delta\; \mathrm{log}\;{Y}_{it}+{\beta }_{2}\Delta \Delta\; \mathrm{log}\;{P}_{it}+{\beta }_{3}{\left(\frac{s}{y}\right)}_{i, t-1}+{\beta }_{4}{R}_{it}^{*}+{\beta }_{5}{\Delta U}_{it}+{\beta }_{6}S{S}_{it}+{\beta }_{7}\;OL{D}_{it}+{\beta }_{8}P{R}_{it}+{\beta }_{9}{GS}_{it}+{\beta }_{10}{HS}_{it}+\sum\limits _{j=1}^{20}{d}_{j}{D}_{j}+{u}_{2,it}.$$

Most variables are the same as in Eq. (1). The three exceptions are unanticipated income growth, ${\Delta \Delta logY}_{it}$, which is now the second difference of the log of income, unanticipated inflation, ${\Delta \Delta logP}_{it}$, now the second difference of the log of the implicit expenditure deflator, and the real interest rate, ${R}_{it}^{*}$, now the nominal rate minus the lagged inflation rate. The private final consumption-expenditure deflator index (OECD 2023c) is the basis for the calculation of the inflation rate. As in Eq. (1), the nominal interest rate is either the long- or the short-run rate.

To analyze the general effect of negative interest rates, two separate dummies were created. The first dummy, ${\mathcal{R}}_{it}^{l}$, equals 1 when the long-term interest rate is negative for country $i$ in year $t$ and 0 otherwise. The second dummy, ${\mathcal{R}}_{it}^{s}$, was constructed similarly using the short-term interest rates. The static- and the constant-expectation hypotheses then form the basis for Eqs. (3) and (4):

3 $${\left(\frac{s}{y}\right)}_{it}={\beta }_{1}\Delta\; \mathrm{log}\;{Y}_{it}+{\beta }_{2}\Delta\; \mathrm{log}\;{P}_{it}+{\beta }_{3}{\left(\frac{s}{y}\right)}_{i,t-1}+{\beta }_{4}{R}_{it}+{\beta }_{5}{\Delta U}_{it}+{\beta }_{6}S{S}_{it}+{\beta }_{7}\;OL{D}_{it}+{\beta }_{8}P{R}_{it}+{\beta }_{9}{GS}_{it}+{\beta }_{10}{HS}_{it}+{\sum }_{j=1}^{20}{d}_{j}{D}_{j}+{u}_{3,it}$$

and

4 $${\left(\frac{s}{y}\right)}_{it}={\beta }_{1}\Delta \Delta\; \mathrm{log}\;{Y}_{it}+{\beta }_{2}\Delta \Delta\; \mathrm{log}\;{P}_{it}+{\beta }_{3}{\left(\frac{s}{y}\right)}_{i,t-1}+{\beta }_{4}{R}_{it}^{*}+{\beta }_{5}{\Delta U}_{it}+{\beta }_{6}S{S}_{it}+{\beta }_{7}OL{D}_{it}+{\beta }_{8}P{R}_{it0}+{\beta }_{9}{GS}_{it}+{\beta }_{10}{HS}_{it}+{\beta }_{11}{\mathcal{R}}_{it}+\sum\limits _{j=1}^{20}{d}_{j}{D}_{j}+{u}_{4,it,}$$

respectively, where ${\mathcal{R}}_{it}$ is either ${\mathcal{R}}_{it}^{l}$ or ${\mathcal{R}}_{it}^{s}$.

The specific effects of negative interest rates on saving behavior were studied by creating interaction terms of the negative interest rates dummies ${\mathcal{R}}_{it}$ with each of the explanatory variables. This makes it possible to determine whether negative interest rates enforce or weaken each determinant of savings behavior. With ${\mathcal{R}}_{it}$ either ${\mathcal{R}}_{it}^{l}$ or ${\mathcal{R}}_{it}^{s}$, and using the constant- and static-expectation hypotheses, respectively, this implies four additional estimations of Eqs. (5) and (6):

5 $${\left(\frac{s}{y}\right)}_{it}={\beta }_{1}\Delta\; \mathrm{log}\;{Y}_{it}+{\beta }_{1}^{*}{\mathcal{R}}_{it}\Delta\; \mathrm{log}\;{Y}_{it}+{\beta }_{2}\Delta\; \mathrm{log}\;{P}_{it}+{\beta }_{2}^{*}{\mathcal{R}}_{it}\Delta\; \mathrm{log}\;{P}_{it}+{\beta }_{3}{\left(\frac{s}{y}\right)}_{i,t-1}+{\beta }_{3}^{*}{\mathcal{R}}_{it}{\left(\frac{s}{y}\right)}_{i, t-1}+{\beta }_{4}{R}_{it}+{\beta }_{4}^{*}{{\mathcal{R}}_{it}R}_{it}+{\beta }_{5}{\Delta U}_{it}+{\beta }_{5}^{*}{\mathcal{R}}_{it}{\Delta U}_{it}+{\beta }_{6}S{S}_{it}+{\beta }_{6}^{*}{\mathcal{R}}_{it}S{S}_{it}+{\beta }_{7}OL{D}_{it}+{\beta }_{7}^{*}{\mathcal{R}}_{it}OL{D}_{it}+{\beta }_{8}P{R}_{it}+{\beta }_{8}^{*}{\mathcal{R}}_{it}P{R}_{it}+{\beta }_{9}{GS}_{it}+{\beta }_{9}^{*}{{\mathcal{R}}_{it}GS}_{it}+{\beta }_{10}{HS}_{it}+{\beta }_{10}^{*}{{\mathcal{R}}_{it}HS}_{it}+\sum\limits _{j=1}^{20}{d}_{j}{D}_{j}+{u}_{5,it}$$

and

6 $${\left(\frac{s}{y}\right)}_{it}={\beta }_{1}\Delta \Delta \;\mathrm{log}\;{Y}_{it}+{\beta }_{1}^{*}{\mathcal{R}}_{it}\Delta \Delta \;\mathrm{log}\;{Y}_{it}+{\beta }_{2}\Delta \Delta\; \mathrm{log}\;{P}_{it}+{\beta }_{2}^{*}{\mathcal{R}}_{it}\Delta \Delta \;\mathrm{log}\;{P}_{it}+{\beta }_{3}{\left(\frac{s}{y}\right)}_{i,t-1}+{\beta }_{3}^{*}{\mathcal{R}}_{it}{\left(\frac{s}{y}\right)}_{i, t-1}+{\beta }_{4}{R}_{it}^{*}+{\beta }_{4}^{*}{{\mathcal{R}}_{it}{R}_{it}^{*}}_{it}+{\beta }_{5}{\Delta U}_{it}+{\beta }_{5}^{*}{\mathcal{R}}_{it}{\Delta U}_{it}+{\beta }_{6}S{S}_{it}+{\beta }_{6}^{*}{\mathcal{R}}_{it}S{S}_{it}+{\beta }_{7}OL{D}_{it}+{\beta }_{7}^{*}{\mathcal{R}}_{it}OL{D}_{it}+{\beta }_{8}P{R}_{it}+{\beta }_{8}^{*}{\mathcal{R}}_{it}P{R}_{it}+{\beta }_{9}{GS}_{it}+{\beta }_{9}^{*}{{\mathcal{R}}_{it}GS}_{it}+{\beta }_{10}{HS}_{it}+{\beta }_{10}^{*}{{\mathcal{R}}_{it}HS}_{it}+\sum\limits _{j=1}^{20}{d}_{j}{D}_{j}+{u}_{6,it.}$$

Note that Koskela and Virén (1983), Nicolescu-Aron and Mihăescu (2012), Aizenman et al. (2019), El Mekkaoui de Freitas and Martins (2014), and Fredriksson and Staal (2021) used similar specifications in their analysis. This enables relating the findings of this paper, especially for Eqs. (1) and (2), directly to the findings in these papers. The regression models include country-specific intercepts, capturing country heterogeneity. These fixed-effects models (FEM) are estimated using ordinary least squares (OLS).

## Regression Results

The specifications in Eqs. (1) and (2) do not include dummy variables for, or interaction terms with, negative interest rates and are the basis for comparing the rest of the specifications. Table 2 presents the estimation outcomes for these basic specifications. In line with disequilibrium- savings theory, unanticipated income, unanticipated inflation and the lagged savings ratio all have a positive and statistically significant effect on the savings ratio. Koskela and Virén (1983) and Fredriksson and Staal (2021) presented results similar to those of Aizenman et al. (2019) for unanticipated income changes, Nicolescu-Aron and Mihăescu (2012) for unanticipated inflation, and Aizenman et al. (2019) and El Mekkaoui de Freitas and Martins (2014) for the lagged saving rates. The interest rates have either a significant negative (long-term rate) or positive (short-term rate) effect, reflecting the ambiguous theoretical prediction of opposite income and substitution effects. Nicolescu-Aron and Mihăescu (2012) and El Mekkaoui de Freitas and Martins (2014) both presented evidence for a statistically significant positive effect, while the interest rate remained insignificant in Koskela and Virén (1983) and Fredriksson and Staal (2021).

**Table 2:** Factors of saving behavior: basic specifications

Variables		[1] Saving ratio $\left(s/y\right)$ OLS FEM	[1] Saving ratio $\left(s/y\right)$ OLS FEM	[2] Saving ratio $\left(s/y\right)$ OLS FEM	[2] Saving ratio $\left(s/y\right)$ OLS FEM
	Dependent				
Explanatory					
Unanticipated income change $\left(\Delta \mathrm{log}Y\right)$ or $\left(\Delta \Delta \mathrm{log}Y\right)$		0.453*** (0.095)	0.375*** (0.100)	0.096 (0.064)	0.076 (0.065)
Unanticipated inflation $\left(\Delta \mathrm{log}P\right)$ or $\left(\Delta \Delta \mathrm{log}P\right)$		0.387* (0.216)	0.707*** (0.240)	0.910*** (0.201)	0.587*** (0.211)
Lagged saving rate $\left(s/y\right)$		0.619*** (0.048)	0.615*** (0.048)	0.623*** (0.050)	0.611*** (0.051)
Long-term interest rate $\left(R\right)$ or $\left({R}^{*}\right)$		-0.204* (0.109)		-0.144 (0.090)	
Short-term interest rate $\left(R\right)$ or $\left({R}^{*}\right)$			0.332*** (0.094)		0.290*** (0.089)
First difference unemployment $\left(\Delta U\right)$		0.579*** (0.112)	0.505*** (0.112)	0.387*** (0.117)	0.357*** (0.118)
Social security $\left(SS\right)$		-0.299** (0.117)	-0.353*** (0.124)	-0.315*** (0.119)	-0.429*** (0.121)
Old-age dependency ratio $\left(OLD\right)$		0.150** (0.066)	0.353*** (0.056)	0.229*** (0.057)	0.359*** (0.054)
Participation rate 65+ $\left(PR\right)$		0.042 (0.059)	0.096* (0.057)	0.046 (0.060)	0.091 (0.058)
Government budget surplus $\left(GS\right)$		-0.257*** (0.047)	-0.298*** (0.049)	-0.278*** (0.048)	-0.307*** (0.050)
Health expenditure $\left(HS\right)$		0.171 (0.131)	0.190 (0.132)	0.187 (0.134)	0.213 (0.134)
Observations (*N*)		400	380	380	361
${R}^{2}$		0.812	0.821	0.816	0.825

Standard errors in parenthesis. * Significant at the 10% level ** Significant at the 5% level *** Significant at the 1% level. OLS FEM: Ordinary least squares fixed effects model. Estimates include country dummies (not reported). [3] based on 2001–2020, [2] on 2002–2020. Source: Own calculations using data from OECD (2023a, 2023b, 2023c, 2023d, 2023e, 2023f, 2023g, 2023h, 2023i, 2023j, 2023k)

Income uncertainty, measured as the first difference of unemployment, has a statistically significant positive effect on the savings ratio, as predicted by the permanent income theory. Similar evidence of the positive and significant effects of income uncertainty was presented in Koskela and Virén (1983) and Fredriksson and Staal (2021). Contrary to the predictions based on the permanent-income and life-cycle theories, the old-age dependency ratio has a statistically significant positive effect on the savings ratio. Fredriksson and Staal (2021) also presented evidence for a significant positive effect. El Mekkaoui de Freitas and Martins (2014) found the predicted significant negative effect, while Koskela and Virén (1983), Nicolescu-Aron and Mihăescu (2012) and Aizenman et al. (2019) all found no statistically significant effect. The sometimes statistically significant positive effects of the participation rate also runs counter to theory. Fredriksson and Staal (2021) had similar results, while Koskela and Virén (1983) identified a negative but insignificant effect. The negative and significant effect of a government budget surplus confirms a Ricardian equivalence effect. El Mekkaoui de Freitas and Martins (2014) and Aizenman et al. (2019) presented similar statistically significant evidence. Health expenditure has an insignificant effect. Nicolescu-Aron and Mihăescu (2012) and Aizenman et al. (2019) found a negative and significant effect.

To study the overall effect of negative interest rates, both Eqs. (3) and (4) included a dummy variable for negative interest rates. Table 3 presents the estimation results. In all specifications, the negative interest rate dummies have a statistically significant positive effect on the saving ratio. These increases in the savings ratio are also significant from an economic perspective. The average savings ratio in the sample is 5.713. The estimated parameters for the negative interest rates dummies are between 0.692 and 3.083, suggesting that the savings ratio increases with a factor between 1.121 and 1.539. The other estimated parameters had similar implications as the ones presented for the basic specifications in Eqs. (1) and (2). There is still significant support for the disequilibrium-savings theory given the estimated parameters for unanticipated income change, unanticipated inflation, and the lagged savings rate. Income uncertainty, approximated as the first difference of the unemployment rate, still has a positive effect on savings, in line with the permanent-income theory prediction. The effect of social security spending on savings remains negative, as predicted by the permanent-income and the life-cycle theories. However, the estimated parameters for the old age dependency ratio and the participation rate are again not in line with the permanent-income and the life-cycle hypotheses. The long-term interest rate no longer has a significant effect, while there is still a positive effect of short-term interest on the savings ratio. The Ricardian equivalence effect remains significant, given the estimates of the government surplus parameters. Finally, health expenditures now has a weakly significant positive instead of the expected negative effect on the savings ratios.

**Table 3:** Negative interest rates: overall effect

Variables		[3] Saving ratio $\left(s/y\right)$ OLS FEM	[3] Saving ratio $\left(s/y\right)$ OLS FEM	[4] Saving ratio $\left(s/y\right)$ OLS FEM	[4] Saving ratio $\left(s/y\right)$ OLS FEM
	Dependent				
Explanatory					
Unanticipated income change $\left(\Delta \mathrm{log}Y\right)$ or $\left(\Delta \Delta \mathrm{log}Y\right)$		0.448*** (0.091)	0.346*** (0.099)	0.129** (0.061)	0.075 (0.064)
Unanticipated inflation $\left(\Delta \mathrm{log}P\right)$ or $\left(\Delta \Delta \mathrm{log}P\right)$		0.483** (0.208)	0.614** (0.239)	0.834*** (0.194)	0.573*** (0.208)
Lagged saving rate $\left(s/y\right)$		0.602*** (0.046)	0.626*** (0.048)	0.606*** (0.049)	0.623*** (0.050)
Long-term interest rate $\left(R\right)$ or $\left({R}^{*}\right)$		-0.145 (0.105)		-0.086 (0.088)	
Short-term interest rate $\left(R\right)$ or $\left({R}^{*}\right)$			0.387*** (0.085)		0.303*** (0.089)
First difference unemployment $\left(\Delta U\right)$		0.479*** (0.109)	0.544*** (0.111)	0.312*** (0.113)	0.394*** (0.118)
Social security $\left(SS\right)$		-0.284** (0.112)	-0.331*** (0.122)	-0.303*** (0.115)	-0.425*** (0.120)
Old-age dependency ratio $\left(OLD\right)$		0.096 (0.064)	0.265*** (0.063)	0.175*** (0.056)	0.255*** (0.064)
Participation rate 65 + $\left(PR\right)$		0.041 (0.057)	0.090 (0.056)	0.042 (0.058)	0.082 (0.058)
Government budget surplus $\left(GS\right)$		-0.246*** (0.045)	-0.302*** (0.048)	-0.266*** (0.046)	-0.310*** (0.050)
Health expenditure $\left(HS\right)$		0.213* (0.126)	0.256* (0.132)	0.227* (0.129)	0.278** (0.134)
Negative interest rate dummy $\left(\mathcal{R}\right)$		3.274*** (0.575)	1.140*** (0.381)	3.146*** (0.584)	1.075*** (0.372)
Observations (*N*)		400	380	380	361
${R}^{2}$		0.827	0.825	0.830	0.829

Standard errors in parenthesis. * Significant at the 10% level ** Significant at the 5% level *** Significant at the 1% level. OLS FEM: Ordinary least squares fixed effects model. Estimates include country dummies (not reported). [3] based on 2001–2020, [4] on 2002–2020. Source: Own calculations using data from OECD (2023a, 2023b, 2023c, 2023d, 2023e, 2023f, 2023g, 2023h, 2023i, 2023j, 2023k)

Table 4 presents the estimates for Eqs. (5) and (6) and allows for a more specific study of how negative interest rates change the effects of the explanatory variables on the savings ratio. Disequilibrium-savings theory predicts that unanticipated income changes, unanticipated inflation and lagged savings should have positive effects. The interaction term of unanticipated income change and negative interest rate has the expected positive and significant effect in one of the four specifications. The interaction term of unanticipated inflation and the negative interest rate dummy has a dissimilar effects in the four estimations. However, two of the interaction term lagged savings are statistically significant and negative. Thus, negative interest rates decreases the positive effect of this variable, weakening the evidence in favor of the disequilibrium-savings theory. The interaction term of the government budget surplus and the dummy for negative interest rates are negative and statistically significant. Thus, the Ricardian equivalence effect is stronger when interest rates are negative. The effects of negative interest rates on the predictive power of the permanent-income and life-cycle theories are mixed. The interaction term of income uncertainty (i.e., the first difference of unemployment) is negative, increasing the predictive power of these theories in times of negative interest rates. The estimated interaction terms of negative interest rates with social security spending the old-age dependency ratio, participation rate of the elderly and health expenditure, however, indicate a decrease in the predictive power of these theories in times of negative interest rates.

**Table 4:** Negative interest rates: specific effects

Variables		[5] Saving ratio $\left(s/y\right)$ OLS FEM	[5] Saving ratio $\left(s/y\right)$ OLS FEM	[6] Saving ratio $\left(s/y\right)$ OLS FEM	[6] Saving ratio $\left(s/y\right)$ OLS FEM
	Dependent				
Explanatory					
Unanticipated income change $\left(\Delta \mathrm{log}Y\right)$ or $\left(\Delta \Delta \mathrm{log}Y\right)$ interaction with $\mathcal{R}$		0.412*** (0.088) 3.345* (1.866)	0.457*** (0.097) -0.104 (0.272)	0.130** (0.059) -0.999 (1.268)	0.189*** (0.064) -0.192 (0.189)
Unanticipated inflation $\left(\Delta \mathrm{log}P\right)$ or $\left(\Delta \Delta \mathrm{log}P\right)$ interaction with $\mathcal{R}$		0.521** (0.203) -9.267*** (3.407)	0.713*** (0.223) -0.026 (0.086)	0.788*** (0.185) 1.455* (0.868)	0.568*** (0.196) 0.530 (1.143)
Lagged saving rate $\left(s/y\right)$ interaction with $\mathcal{R}$		0.627*** (0.046) -0.186 (0.620)	0.634*** (0.046) -0.276*** (0.103)	0.636*** (0.047) -1.198*** (0.457)	0.652*** (0.050) -0.079 (0.070)
Long-term interest rate $\left(R\right)$ or $\left({R}^{*}\right)$ interaction with $\mathcal{R}$		-0.142 (0.102) -49.242*** (16.918)		-0.072 (0.084) -3.562** (1.554)	
Short-term interest rate $\left(R\right)$ or $\left({R}^{*}\right)$ interaction with $\mathcal{R}$			0.265*** (0.088) -4.745*** (1.673)		0.262*** (0.085) -0.700** (0.346)
First difference unemployment $\left(\Delta U\right)$ interaction with $\mathcal{R}$		0.502*** (0.104) -10.581** (4.132)	0.542*** (0.112) 0.097 (0.298)	0.356*** (0.109) -6.261*** (2.297)	0.427*** (0.118) 0.073 (0.351)
Social security $\left(SS\right)$ interaction with $\mathcal{R}$		-0.227** (0.109) 2.234** (0.905)	-0.324*** (0.119) -0.332** (0.146)	-0.272** (0.111) -0.360 (0.586)	-0.419*** (0.120) -0.268* (0.145)
Old-age dependency ratio $\left(OLD\right)$ interaction with $\mathcal{R}$		0.105* (0.063) -1.093*** (0.368)	0.218*** (0.062) -0.101 (0.095)	0.177*** (0.054) -0.382 (0.268)	0.205*** (0.065) 0.087 (0.088)
Participation rate 65+ $\left(PR\right)$ interaction with $\mathcal{R}$		0.042 (0.054) 3.537*** (0.901)	0.071 (0.052) 0.139 (0.095)	0.035 (0.055) 0.813** (0.340)	0.074 (0.055) 0.107 (0.097)
Government budget surplus $\left(GS\right)$ interaction with $\mathcal{R}$		-0.216*** (0.044) -1.634*** (0.298)	-0.225*** (0.045) -0.707*** (0.102)	-0.227*** (0.045) -0.928*** (0.311)	-0.239*** (0.047) -0.698*** (0.117)
Health expenditure $\left(HS\right)$ interaction with $\mathcal{R}$		0.284** (0.121) -4.719 (3.015)	0.272** (0.123) 0.992*** (0.303)	0.253** (0.125) 1.901 (1.619)	0.343*** (0.126) -0.074 (0.078)
Observations (*N*)		400	380	380	361
${R}^{2}$		0.858	0.863	0.849	0.856

Standard errors in parenthesis. * Significant at the 10% level ** Significant at the 5% level *** Significant at the 1% level. OLS FEM: Ordinary least squares fixed effects model. Estimates include country dummies (not reported). $\mathcal{R}$: negative interest rate dummy. [5] based on 2001–2020, [6] on 2002–2020. Source: Own calculation using data from OECD (2023a, 2023b, 2023c, 2023d, 2023e, 2023f, 2023g, 2023h, 2023i, 2023j, 2023k)

## Concluding Remarks

Based on a panel of 20 countries covering the years 2000 to 2020, this paper analyzes determinants of savings behavior using panel data. This data set is generated from the OECD website. From the fixed-effects least-squares regression analysis, it follows that negative interest rates strengthen the Ricardian equivalence effect while the effect on the empirical validity of the disequilibrium-savings, permanent-income and life-cycle theories is mixed. Finally, the analysis suggests that negative interest rates increase savings in a statistically, as well as in an economically, significant way. The significant and robust evidence that negative interest rates increase household’s savings implies that a negative interest rate policy is counter-productive as an instrument to stimulate households’ consumption expenditures.

As this is, to my knowledge, one of the first papers considering the effects of negative interest rates on saving behavior, there remain many possibilities for further research such as the use of an alternative research approach, e.g., one based on a vector autoregressive model. Other ways to extend the research include accounting for the effects of other forms of unconventional monetary policy, like quantitative easing, on household savings. A deeper theoretical and empirical understanding is necessary for the evaluation of unconventional monetary policy.

## Data Availability

A data availability statement is now in the second sentence of the Concluding Remarks.

## Footnotes

1 Australia, Austria, Belgium, Canada, Czechia, Germany, Denmark, Spain, Finland, France, the United Kingdom, Hungary, Ireland, Italy, the Netherlands, Norway, Portugal, Slovakia, Sweden, and the United States of America.
